# The Chatbots Are Invading Us: A Map Point on the Evolution, Applications, Opportunities, and Emerging Problems in the *Health Domain*

**DOI:** 10.3390/life13051130

**Published:** 2023-05-05

**Authors:** Daniele Giansanti

**Affiliations:** Centre Tisp, Istituto Superiore di Sanità, 00161 Roma, Italy; daniele.giansanti@iss.it; Tel.: +39-06-49902701

**Keywords:** chatbot, health, health domain, artificial intelligence

## Abstract

The inclusion of chatbots is potentially disruptive in society, introducing opportunities, but also important implications that need to be addressed on different domains. The aim of this *study* is to examine chatbots in-depth, by mapping out their technological evolution, current usage, and potential applications, opportunities, and emerging problems within the *health domain*. The study examined three *points of view*. The *first point of view* traces the technological evolution of chatbots. The *second point of view* reports the fields of application of the chatbots, giving space to the expectations of use and the expected benefits from a cross-domain point of view, also affecting the *health domain.* The *third and main point of view* is that of the analysis of the state of use of chatbots in the health domain based on the scientific literature represented by systematic reviews. The overview identified the topics of greatest interest with the opportunities. The analysis revealed the need for initiatives that simultaneously evaluate multiple domains all together in a synergistic way. Concerted efforts to achieve this are recommended. It is also believed to monitor both the process of osmosis between other sectors and the *health domain*, as well as the chatbots that can create psychological and behavioural problems with an impact on the *health domain.*

## 1. Introduction

The world has recently witnessed the diffusion of the technological phenomenon of chatbots [1,2,3,4]. This phenomenon is simultaneously attracting and worrying public opinion, scholars and stakeholders. The attraction is due to the rapid diffusion, the easy accessibility, and the opportunities that chatbots, increasingly integrated with artificial intelligence, seems to offer. However, it is worrying that such rapid diffusion and easy accessibility have not been adequately accompanied by robust reflections on the impact they have on many domains of public life, from the social to the ethical and regulatory.

A chatbot can be defined as:

1. *A computer program designed to have a conversation with a human being, especially over the internet* (https://dictionary.cambridge.org/dictionary/english/chatbot [1]);
2. *A computer program in the form of a virtual e-mail correspondent that can reply to messages from computer users* (https://www.dictionary.com/browse/chatbot [2]; https://www.collinsdictionary.com/dictionary/english/chatbot [3]);
3. A bot that is designed to converse with human beings. Bot: computer program or character (as in a game) designed to mimic the actions of a person (https://www.merriam-webster.com/dictionary/chatbot [4]).

The introduction of artificial intelligence (AI) has inspired further stimulating scientific debates regarding its large-scale application, including in the world of healthcare [5]. An example of this is represented by the chatGPT tool [6], which has recently become widespread, rapidly attracting scientific attention to its potential and implications in its applications in social life [7] and in the health domain [8]. On the other hand, facing the challenges of integrating technologies with AI is an open and current challenge with opportunities, challenges, and bottlenecks to overcome, affecting several domains [9,10], and also affecting learning processes and ethics [11].

The inclusion of chatbots is potentially disruptive in society, introducing opportunities but also important implications that need to be addressed on different domains.

The aim of this analysis is to examine chatbots in depth by mapping out their technological evolution, current usage, and potential applications within the health domain.

The sub-aims are:

- To determine the historical development of chatbot technology and its evolution over time;
- To analyze the current usage patterns of chatbots within different fields;
- To identify the key features, applications and opportunities of chatbots that are specific to the *health domain*;
- To identify the potential problems and bottlenecks in the *health domain*.

## 2. Methods

The study was arranged into three points of view. 

The *first point of view* traces the technological evolution of chatbots starting from the first pioneering experiences. 

The *second point of view* reports the fields of application of the chatbots both from the point of view of categorization and of the sector of use, also giving space to the expectations of use and the expected benefits from a cross-domain point of view, also impacting the *health domain*. 

The *third and main point* of view is that of the analysis of the state of use of chatbots in the health domain based on the scientific literature represented by systematic reviews. We decided to analyze the systematic reviews, the highest level of evidence in healthcare, because they are capable of providing a comprehensive evaluation of a particular topic by identifying and analyzing all the available primary research studies. This summary of evidence can help detect the principal patterns of interest and highlight areas where additional research is needed or where current research is insufficient to support clinical decisions, in this case, in the clinical domain.

The overview related to the first and second point of views was based on targeted searches on Google and Google Scholar. 

The overview related to the third point of view followed a targeted search on PubMed by means of a properly settled composite key.

The overview, as a whole followed the ANDJ checklist, a standardized checklist for the structure of a narrative review.

This overview was carefully crafted with a consideration of five parameters (N1–N5) that have been evaluated on a scale ranging from one (minimum) to five (maximum). The parameters are as follows:

N1: Clarity of introduction and rationale for the review.

N2: Appropriateness of review design.

N3: Clear description of methods.

N4: Clear presentation of results.

N5: Justification of conclusions based on results.

N6: Full disclosure of potential conflicts of interest by authors.

These parameters have been thoughtfully selected to ensure the comprehensiveness and quality of this overview. All selected elements must receive a score of at least three on all parameters in order to be included.

## 3. Results

### 3.1. An Overview of the Evolution of Technology

The origin of chatbots [12] can probably be attributed to Alan Turing’s 1950s vision of intelligent machines. Artificial intelligence, the basis of chatbots, has therefore, developed these tools. We can summarize this evolution in brief [12]. The first chatbot named *ELIZA*, created in 1966, simulated a psychotherapist’s function, repeating the users’ sentences in an interrogative form. Its ability to communicate was limited; however, it can be considered a source of inspiration for further evolutions [13,14]. In 1972, *PARRY* was introduced. It acted as a patient with schizophrenia and defined its responses based on a system of assumptions and ‘‘emotional responses’’ [15]. AI was firstly used in the domain of the chatbots with the introduction of *Jabberwacky* in 1988. CleverScript was used in this system. It was a language based on spreadsheets, which was useful in the development of chatbots. This system was able to respond based on previous answers. It was limited in speed and number of users [16]. The term *CHATTERBOT* was introduced *in 1991*. It was an artificial player with a primary function of chatting [17]. *Dr. Sbaitso* appeared in 1992 [18]. It played the role of a psychologist seemingly without showing complications in its interactions with users [18]. *ALICE* was a further step forward in the world of chatbots. It used the artificial intelligence markup language. It performed better compared to ELIZA [19]. *SmarterChild* was introduced in *2001*. It was integrated with Messengers. This chatbot, for the first time, could help people with useful daily tasks using large databases with information related to movie times, sports, weather, and other information [20]. The chatbot using AI made another important step forward with the introduction of the smart personal voice assistants between 2010 and 2020. They were capable of understanding vocal commands and informative tasks. *Apple Siri, IBM Watson, Google Assistant, Microsoft Cortana, and Amazon Alexa* are the most popular voice assistants [21,22,23,24,25,26,27]. Early in 2016, a further evolution in AI technology radically changed the communicative interaction between users and manufactures. Social media platforms allowed developers to create chatbots that allowed the clients to complete specific tasks using their own messaging applications. At the end of 2016, the chatbots covered a wide range of applications ranging from entertainment to healthcare, including marketing, education, generalized support, cultural heritage, and much more. Moreover, the Internet of Things allowed new fields of application for chatbots; they played the role of connectors and mediators of “smart objects” [28]. See for example *Microsoft XiaoIce*, an AI-based chatbot with the role of satisfying the human need for sociability [29]. At the end of this process of evolution, the way of engaging in discussion with a chatbot was completely different from the *ELIZA* chatbot. Today, a chatbot is capable of sharing personal thoughts along with family drama events.

### 3.2. Exploring the Wide Range of Applications

Chatbots have many desirable characteristics that potentially make them an ideal interlocutor to interact and work with [30,31,32]. In fact, chatbots are potentially able to work efficiently 24/7. They can be customized with user data to try to create a better collaboration, compliance, and, ultimately, a *real virtual symbiosis* with the user. They also have the desirable IT property of scalability, being able to handle a large volume of data and requests/interactions simultaneously. Finally, they allow for savings in resources, being able, through their automatisms, to autonomously carry out tasks, resulting in economic savings in the management of the processes.

Today, chatbots have several applications.

For example, they can be applied, for example, to the following sectors [30,31,32]:

- *Customer service*: Chatbots can be used as virtual assistants to provide 24/7 customer support, answer frequently asked questions, resolve issues and handle customer complaints;
- *Sales and marketing*: Chatbots can assist customers in making purchases, provide product recommendations and cross-sell or upsell other products and services;
- *Healthcare:* Chatbots can provide health-related information, answer medical questions, assist patients in scheduling appointments and in other activities, such as refilling prescriptions, virtual triages, and self-monitoring.
- *Education:* Chatbots can help students with homework assignments, providing study documents and answering, for example, to specific questions.
- *Finance:* Chatbots can assist customers with banking tasks such as checking account balances, transferring money and paying invoices;
- *Travel and tourism*: Chatbots can help travelers plan their trips, book flights and hotels and provide information on tourist attractions;
- *Entertainment:* A chatbot can provide games or other tools to give fun time.
- *Industry:* Chatbots can provide suggestions, give general and specific assistance, support customers on specific products, checking inventory levels and providing assistance with returns and exchanges.

By analyzing the applications under a broader and cross-domain perspective independent of the categories, it can be noted that chatbots today can find up to 37 applications [33]. Population surveys have shown that chatbots are most expected to [33]: quickly respond to an emergency (37%), solve a complex problem (35%) and Respond quickly (35%). As far as the expected benefits are concerned, the following are mostly expected [34]: having access to a 24/7 service (64%), obtaining quick answers (55%) and answering elementary questions (55%). As can be seen, these “expectations” and “expected benefits” of the different categories identified seem to overlap, and touch many sectors, including the very important one of *healthcare*. In fact, in the healthcare domain, having a rapid response in the event of an emergency, in a 24 h mode, is strategic *in emergency medicine and intensive hospitalization medicine*.

### 3.3. The Chatbots in the Health Domain: Applications, Opportunities, Open Challenges, and Problems

The chatbots are increasingly showing several applications in the *health domain*. Diverse keywords [35,36,37,38,39,40,41,42,43,44,45,46,47,48,49,50,51,52,53,54,55] are associated with the concept of the chatbot in healthcare; among the most frequent we find: *patient engagement, clinical support, mental health, health monitoring, patient education, appointment scheduling, symptom checking, chronic disease management, triage, remote monitoring, telemedicine, health coaching and emergency response.*

Chatbots can be used in several fields in the health domain [35,36,37,38,39,40,41,42,43,44,45,46,47,48,49,50,51,52,53,54,55]: (a) As a tool for answering frequently asked questions [39]; (b) For the collection of data and patient details [39,42,43]; (c) To support patients finding a doctor or a specific service, on managing appointments, and on the medication dispensing procedure [42]; (d) As an interactive guide to the management of self-assessment and symptom control [39,42]; (e) As a tool to guide an interactive triage, applicable in the case of an emergency as well [36]; (f) In telehealth, digital health applications, and remote monitoring [37,39,40,42,43]. (g) In the learning process, in the construction of scientific knowledge and in supporting scientific dissemination [35,38]; (h) In mental health applications [37,38]; (i) For physical wellness and health coaching [40].

From a general point of view, the use of these tools has the potential to lighten the hospital and care facility load, decentralizing many of the activities, allowing them to be carried out in a remote mode, something that during a situation such as the COVID-19 pandemic better protects all the actors involved. The patients can be more responsible, self-diagnose independently, and invited and supported to take better care of themselves also in relation to the wellness and psychological aspects. 

A search was performed on PubMed with the following composite key

*(chatbot[Title/Abstract]) AND ((health [Title/Abstract]) OR (healthcare[Title/Abstract]) OR (health domain[Title/Abstract])).*

The key showed the evolution of scientific dissemination in this area. Since 2010, *370* papers have been published, including 19 systematic reviews.

The research highlights a terrific growth in the volume of publications in the last three years, coinciding with the outbreak of the pandemic, with 340 of the papers were published from 2020 to 2022, which is *91.9%* of the total papers published, and the number of papers published in *2022* is *117*, which is *31.62%* of the total papers published.

We decided to analyze the systematic reviews [37,40,45,49,51,52,54,56,57,58,59,60,61,62,63,64,65,66,67] to detect the principal patterns of interest and highlight areas where additional research is needed or where current research is insufficient to support clinical decisions to introduce these tools in the clinical routine.

The analysis of the systematic review allowed us to detect five areas of interest, which are as follows:

- *Application of chatbots in mental health;*
- *Application of chatbots in the domain of the addiction;*
- *Application of chatbots in the domain of the chronic disease;*
- *Application of chatbots in the domain of the wellness and fitness;*
- *Heterogeneous applications in the health domain;*
- *Technology assessment.*

#### 3.3.1. Application of Chatbots in Mental Health

Five studies have investigated the application of chatbots in *mental health* [45,61,65,66,67]. Lim et al. [61] reviewed the effectiveness of chatbot-delivered psychotherapy in improving depressive symptoms in adults with depression or anxiety. The review highlighted that chatbot-delivered psychotherapy significantly improved depressive symptoms. The preferred features for the design of chatbots include embodiment, a combination of input and output formats, less than 10 sessions, problem-solving therapy, offline platforms, and different regions of the United States. The study concluded that chatbot-delivered psychotherapy could be an alternative treatment for depression and anxiety, and further high-quality trials were needed to confirm its effectiveness.

Ruggiano et al. [65] identified current commercially available chatbots that were de-signed for use by people with dementia and their caregivers, and assessed their quality in terms of features and content. Although the chatbots were generally found to be easy to use, limitations were noted regarding their performance and programmed content for dialog. The authors concluded that evidence-based chatbots were needed to adequately educate and support people with dementia and their caregivers.

Vaidyam et al. [66] reviewed the use of conversational agents (chatbots or voice as-assistants) in the assessment and treatment of serious mental illnesses, such as depression, anxiety, schizophrenia, and bipolar disorder. The study highlighted positive outcomes for diagnostic quality, therapeutic efficacy, and acceptability. However, certain populations, such as pediatric patients and those with schizophrenia or bipolar disorder, were un-der-represented in the research. The authors recommended the standardization of studies to include measures of patient adherence and engagement, therapeutic efficacy, and clinician perspectives.

Gaffney et al. [67] investigated the use of conversational agent interventions in mental health. The interventions were diverse and targeted a range of mental health problems using various therapeutic orientations. All included studies reported reductions in psychological distress post-intervention, and the controlled studies demonstrated significant reductions in psychological distress compared to inactive control groups. However, the authors concluded that a more robust experimental design was required to demonstrate efficacy and efficiency. 

Hoermann et al. [45] analyzed the feasibility and effectiveness of one-on-one mental health interventions that used chatbots. The interventions showed significant improvements compared to waitlist conditions, but were not superior to the usual treatment. The study also found substantial innovation in the use of trained volunteers and chatbot technologies. However, further research was needed to determine the feasibility of this mode of intervention in clinical practice.

#### 3.3.2. Application of Chatbots in the Domain of the Addiction

The field of addiction was dealt with in three studies [49,57,62].

Aggarwal et al. [49] evaluated the feasibility, efficacy, and characteristics of AI chatbots for promoting health behavior change. The review found that AI chatbots have shown high efficacy in promoting healthy lifestyles, smoking cessation, treatment or medication adherence, and reduction in substance misuse. However, there were mixed results regarding feasibility, acceptability, and usability. Furthermore, the authors concluded that the reported results needed to be interpreted with caution due to limitations in internal validity, insufficient description of AI techniques, and limited generalizability. Future studies should adopt robust randomized control trials to establish definitive conclusions.

He et al. [57] also investigated conversational agents for smoking cessation. The systematic review and meta-analysis found that all studies reported positive effects on cessation-related outcomes. Meta-analyses of randomized controlled trials showed that conversational agents were more effective in promoting abstinence compared to control groups. However, the included studies were diverse in design, and evidence of publication bias was identified. The review also highlighted a lack of theoretical foundations and a need for relational communication in future designs. The standardization of reporting on and designing conversational agents was warranted for a more comprehensive evaluation. Overall, this review provided insights into the potential of conversational agents for smoking cessation and the need for further research and development to improve their effectiveness and acceptability.

Ogilvie et al. [62] researched the use of chatbots in the field of addiction, specifically as supportive agents for those with a substance use disorder. The findings suggested that the corpus of the research in this field is limited, and more research was needed to confidently report on the usefulness of chatbots in this area. While some papers reported a reduction in substance use in participants, caution was advised as expert input was needed to safely leverage existing data and avoid potential harm to the intended audience.

#### 3.3.3. Application of Chatbots in the Domain of the Chronic Disease

Two studies focused on the domain of chronic disease [58,60].

Pernencar et al. [58] studied the field of e-Therapy and mobile apps integrated into healthcare systems. The study reviewed the connection between chatbots with inflammatory bowel disease patients’ healthcare, with the goal of supporting the development of digital products for chronic diseases. The study highlighted that the chatbot technology for chronic disease self-management had high acceptance and usability levels. However, the chatbot ontology still needed strong guidelines for personalizing communication.

Sawad et al. [60] explored different conversational agents used in healthcare for chronic conditions, analyzing their communication technology, evaluation measures, and AI methods. They found that users provided positive feedback about the usefulness, satisfaction, and ease of use of conversational agents. However, there was still insufficient evidence to determine the efficacy of AI-enabled conversational agents for chronic health conditions due to the lack of reporting of technical implementation details.

#### 3.3.4. Application of Chatbots in the Domain of the Wellness and Fitness

Two studies explored the application of chatbots in the domain of wellness and fitness [40,64].

Luo et al. [64] examined the use of conversational agents in promoting physical activity (PA). Conversational agents were found to have moderate usability and feasibility. The authors reported that conversational agents were effective in promoting PA. However, they highlighted the need for further research on the long-term effectiveness and safety of conversational agents in promoting PA, as well as the importance of using evidence-informed theories and addressing user preferences for variety and natural language processing. 

Oh et al. [40] looked at studies evaluating the use of AI chatbots in changing physical activity, healthy eating, weight management behaviors, and other related health out-comes. The study found that chatbot interventions were promising in increasing physical activity but limited in changing diet and weight status. The review reported that the studies had inconsistent outcome assessments on chatbot characteristics. The study recommended standardization of designing and reporting chatbot interventions in the future. Overall, the authors concluded that chatbots might improve physical activity, but more research is needed on their efficacy for diet and weight management/loss.

#### 3.3.5. Heterogeneous Applications in the Health Domain

Five studies analysed multiple applications simultaneously in the *health domain* [37,51,52,56,63].

Milne-Ives et al. [52] discussed the increasing use of conversational agents in healthcare to support a variety of activities, such as behavior change, treatment support, health monitoring, training, triage and screening support. In particular, the review evaluated the effectiveness and usability of these agents and identified the elements that users liked and disliked. The evidence generally reported positive or mixed results for effective-ness, usability and satisfactoriness. However, qualitative feedback highlighted limitations of the agents, and the study design quality was limited. Further research was needed to evaluate the cost-effectiveness, privacy and security of these agents.

Geoghegan et al.’s [51] study reviewed the use of chatbots in the follow-up care of patients who underwent physical healthcare interventions. The included studies analyzed chatbots that were used for monitoring after cancer management, hypertension and asthma, orthopedic intervention, ureteroscopy, and intervention for varicose veins. All chatbots were deployed on mobile devices, and a range of metrics were identified. Importantly, no study examined patient safety. The authors suggested that further investigation was needed to evaluate the acceptability, efficacy and mechanistic evaluation of chatbots in routine clinical care.

Xu et al. [63] reviewed the recent advancements of and current trends in the use of chatbot technology in medicine, particularly in cancer therapy. The article provided a brief historical overview and discussed the design characteristics and the potential uses of chatbots in diagnosis, treatment, monitoring, patient support, workflow efficiency and health promotion. The article also addresses limitations and areas of concern, including ethical, moral, security, technical and regulatory standards. The authors concluded that chatbots have the potential to be integrated into clinical practice by working alongside health practitioners to reduce costs, refine workflow efficiencies and improve patient outcomes. However, they called for further research and interdisciplinary collaboration to advance this technology and improve the quality of care for patients.

Huq et al. [37] investigated the potential benefits of chatbots and conversational agents in improving the quality of life for aged and impaired individuals. The study emphasized the need for further research and development to fill knowledge gaps in remote healthcare and rehabilitation, which could ultimately lead to improved outcomes for patients.

Sallam [56] proposed a review on ChatGPT, an artificial intelligence (AI) chatbot that uses large language models. The review examined the potential benefits and limitations of using ChatGPT in healthcare education, research and practice. The article found that ChatGPT has several potential benefits, including improving scientific writing, enhancing research equity and versatility and improving personalized learning. However, there were also significant concerns surrounding ChatGPT’s use, including ethical, copyright, transparency and legal issues, the risk of bias, plagiarism, lack of originality, cybersecurity issues and inaccurate content. Despite the potential benefits, the review recommended caution when using ChatGPT and other similar tools in healthcare and academia, calling for a code of ethics to guide their responsible use.

#### 3.3.6. Technology Assessment

Technology assessment was investigated in two studies [54,59].

Denecke and May [59] discussed the use of conversational agents (CAs) in healthcare and the lack of a standard procedure to study their usability. The authors conducted a systematic literature review and found that a variety of tools and metrics were used to assess usability, but there was little consistency in the study designs. As a result, they found that it was difficult to compare usability among different CAs. The authors recommended the development of a standardized procedure for evaluating CA usability that can be applied consistently and can be tailored to specific features of individual CAs.

Chattopadhyay et al. [54] investigated the effectiveness of virtual humans (VH) in patient-facing systems. The study also identified two design categories—simple VH and VH augmented with health sensors and trackers. The intervention was mainly delivered using personal computers, and more focused analysis to identify what features of VH interventions contributed toward their effectiveness is needed in the future. Overall, the study offered evidence for the efficacy of VH in patient-facing systems, but further research is required to fully understand their potential benefits.

## 4. Discussion

Chatbots have undergone a terrific evolution through decades of technological innovation. The latest and most impactful advancement is the one we are experiencing, which is represented by artificial intelligence [8,9]. Today, the chatbots can find up to 37 applications [33]. Population expectations of chatbots are many and important. The major expectation of the population with respect to these systems is chatbots’ rapid response in emergency situations [33]. The greatest expected benefit is that of being able to receive 24 h service from these systems. The main “expectations” and the main “expected benefits” of different domains seem to overlap, including the very important one of healthcare. In fact, specifically in healthcare, having a rapid response in the event of an emergency, in a 24 h mode, is strategic in emergency medicine and intensive hospitalization medicine. Additionally, *healthcare* is one of the domains affected by the introduction of these systems. After having retraced, through our study, the evolution of technology, and after having addressed the topic of applications, from which expectations of use and cross-benefits between categories (including the health domain) have emerged, we have focused on the *health domain*. 

An overview on PubMed confirmed a rapid increase in scientific interest in the health domain [68], in line with the perceived general interest [69]. In particular, PubMed showed that in the last three years, a volume of publications equal to 91.2% of total publications on these systems has been published, which, as has been discussed, have existed since 1966 [13]. A substantial contribution to this growth was also made both by the COVID-19 pandemic, which has brought 91 publications since 2020 [70] (see the composite key in the first position of the Box 1 reported below), and by artificial intelligence, which was of interest in 123 studies [71] (see the composite key in the second position of the Box 1 reported below). 

The topics of greatest interest emerged from a search on PubMed based on systematic reviews. These studies concerned two specific systematic reviews on *technology assessment* and systematic reviews analyzing chatbot applications in *mental health, addiction, chronic diseases, wellness and fitness, and, finally, the applications on heterogeneous applications in the health domain and technology assessment.*

The two studies [54,59] on *technology assessment*, even if limited to a few domains, gave us important indications on chatbot technology. 

The first one [59] highlighted the lack of a standard procedure to study usability, with a variety of different tools and metrics used to assess usability with little consistency in the study designs. The authors recommend the development of a standardized procedure for evaluating usability. 

The second [54], while acknowledging the potential effectiveness of these systems, deemed it necessary to further investigate the real requirements that make chatbots effective and the potential benefits. 

The other studies have unanimously highlighted the potential opportunities of these systems in specific applications but have highlighted various *critical issues concerning different single domains.*

The need for more attention on the *domain of ethics* has been recommended, for example in [56], with regard to the applications in healthcare education, research and practice.

The need to deepen the *domain of safety*, to avoid the potentially harmful impact of these systems has been highlighted in [62], with a focus on the domain of drug addiction.

The need for more attention on the *domain of standardization* has been highlighted by three studies [40,49,58]. In [58], the development of specific guidelines was recommended in a *chronicity study*. Reference [49] suggested the standardization of procedures and protocols in the domain of *addiction*. Even in *applications dedicated to fitness and wellness*, the need for standardization has been recalled both in design and reporting [40]. 

Concerns regarding the *domain of efficacy* have been raised in several studies [40,45,51,57,60,61,64,65,67]. An in-depth study of this domain has been suggested in various applications of mental health [45,61,65,67] in the domain of addiction [57], in the field of chronicity [60], in post-intervention medicine [51] and in wellness and fitness [40,64].

The *domain of cybersecurity* has been touched upon to a certain extent by all the studies, but particularly in two studies that have addressed multiple applications simultaneously in the health domain [52,56]. The *domain of interdisciplinarity* was recalled as important in cancer therapy, where this aspect, as is known, is a key factor [63].

The *need to address some domains* together was highlighted in [56]. In this study, dedicated to AI-based chatbots used in healthcare education, research, and practice, concerns were expressed on *ethical aspects, copyright, transparency, legal issues, the risk of bias, plagiarism, lack of originality, cybersecurity issues and inaccurate content*.’

Box 1 **The proposed composite keys.**

*(“chatbot”[Title/Abstract] AND (“health”[Title/Abstract] OR “healthcare”[Title/Abstract] OR “health domain”[Title/Abstract]) AND (“COVID-19”[All Fields] OR “COVID-19”[MeSH Terms] OR “COVID-19 vaccines”[All Fields] OR “COVID-19 vac-cines”[MeSH Terms] OR “COVID-19 serotherapy”[All Fields] OR “COVID-19 nucleic acid test-ing”[All Fields] OR “COVID-19 nucleic acid testing”[MeSH Terms] OR “COVID-19 serological testing”[All Fields] OR “COVID-19 serological testing”[MeSH Terms] OR “COVID-19 test-ing”[All Fields] OR “COVID-19 testing”[MeSH Terms] OR “SARS-CoV-2”[All Fields] OR “SARS-CoV-2”[MeSH Terms] OR “severe acute respiratory syndrome coronavirus 2”[All Fields] OR “ncov”[All Fields] OR “2019 ncov”[All Fields] OR ((“coronavirus”[MeSH Terms] OR “coro-navirus”[All Fields] OR “cov”[All Fields]) AND 2019/11/01:3000/12/31[Date–Publica-tion]))) AND (2020/1/1:2023/4/14[pdat])*

 

*(“chatbot”[Title/Abstract] AND (“health”[Title/Abstract] OR “healthcare”[Title/Abstract] OR “health domain”[Title/Abstract]) AND (“artificial intelligence”[MeSH Terms] OR (“artificial”[All Fields] AND “intelligence”[All Fields]) OR “artificial intelligence”[All Fields])) AND (2020/1/1:2023/4/13[pdat])*

## 5. Recommendation

A statement by Henry Ford reported that “*real progress happens only when the advantages of a new technology become available to everybody*”. The consolidation of technologies based on chatbots is intended to bring benefits to everyone in several areas. 

Among these areas we find the *health domain*, which is strategic since it has to do with the health of citizens.

The overview highlighted a particular increase in scientific interest in this area, which is accompanied, as for all other sectors of employment, by important expectations on the part of the citizen.

The overview also showed, through an analysis of the sectors most addressed by scholars in the *health domain*, that the need to deepen individual domains, such as effectiveness, legal aspects, and standardization, just to name a few, emerged from time to time. 

What is necessary at this point in the evolution of these tools is to develop studies that simultaneously evaluate multiple domains all together in a synergistic way.

*To do this, it is important that scholars, experts, politicians, and stakeholders stimulate and initiate large-scale consensus initiatives that address these issues by considering the different multiple domains of intervention. Concerted actions involving experts, international scientific societies and stakeholders could be useful for tackling these strategic issues more decisively. Initiatives such as studies on health technology assessment or the Consensus Conference are strongly recommended*. 

These initiatives could provide shared documents, including applications, organization models, training, regulations, ethics, and other domains [72,73].

This overview also highlights the cross-domain character of the topic of chatbots; 37 chatbot applications and some expectations have been identified [33,34]. 

There has always been a process of osmosis of technology between various areas and this is applicable to chatbots as well.

What is important, and this is where the stakeholders come into play, is the accurate monitoring of this process, when the process concerns the *health domain*, given that we are dealing with the health of citizens.

Another important aspect is the impact on the *health domain* that a distorted use of these tools as used in other contexts could generate.

There has recently been a discussion on addiction and the psychological impact (and therefore, on the consequences on the *health domain*) that some applications in use in the world of consumption could generate [74,75,76]. For example, *Replika* [74], which allows you to interact with virtual friends, has been banned in some countries, such as Italy, where the guarantor of privacy has banned its use after having identified the risk of behavioral and psychological problems, especially for young people.

Some chatbots allow you to talk to celebrities and others even to the dead, the so-called deadbots [75]. The latter are fed with memories, letters, messages from our loved ones and simulate interaction with the deceased. With these deadbots, important limits are being crossed, and we are entering a world where the implications are psychological, behavioral, and ethical. With these applications, one can enter delicate and special worlds, whose implications that can impact a person in unpredictable ways. In the religious sphere, there are chatbots created in Italy that address the sacred and the afterlife, as they simulate conversations with saints [76]. Other chatbots are venturing into very particular and specific sectors, with the implications that have been highlighted. Making a list would be unthinkable. It is precisely this difficulty that creates the need for activating serious monitoring actions in this field. 

## 6. Conclusions

In conclusion, this study highlights the opportunity and potential of chatbots in the *health domain*. However, the studies carried out have highlighted from time to time the need to investigate issues relating to individual domains. Given the increase in the interest in this area, also driven by the introduction of artificial intelligence, concerted actions that address all related intervention domains simultaneously are recommended. It is also necessary to monitor the osmosis of technologies from other sectors in the *health domain*, which have to do with the health of citizens. The use of some chatbots used in other sectors could affect the mental health of citizens, and therefore, affect the health domain.

## 7. Limitations

This *study*, in relation to its objective, used all the available systematic reviews on PubMed on the topic of chatbots related to the *health domain*. More specific insights on narrow and very particular topics are suggested in future studies using other components of this biomedical database, as well as other national and international databases. 
